# Clinical applicability of deep learning-based respiratory signal prediction models for four-dimensional radiation therapy

**DOI:** 10.1371/journal.pone.0275719

**Published:** 2022-10-18

**Authors:** Sangwoon Jeong, Wonjoong Cheon, Sungkoo Cho, Youngyih Han

**Affiliations:** 1 Department of Health Sciences and Technology, SAIHST, Sungkyunkwan University, Seoul, Korea; 2 Proton Therapy Center, National Cancer Center, Goyang, Korea; 3 Department of Radiation Oncology, Samsung Medical Center, Seoul, Korea; 4 Department of Radiation Oncology, Samsung Medical Center, Sungkyunkwan University School of Medicine, Seoul, Korea; University of Malaya, MALAYSIA

## Abstract

For accurate respiration gated radiation therapy, compensation for the beam latency of the beam control system is necessary. Therefore, we evaluate deep learning models for predicting patient respiration signals and investigate their clinical feasibility. Herein, long short-term memory (LSTM), bidirectional LSTM (Bi-LSTM), and the Transformer are evaluated. Among the 540 respiration signals, 60 signals are used as test data. Each of the remaining 480 signals was spilt into training and validation data in a 7:3 ratio. A total of 1000 ms of the signal sequence (T_s_) is entered to the models, and the signal at 500 ms afterward (P_t_) is predicted (standard training condition). The accuracy measures are: (1) root mean square error (RMSE) and Pearson correlation coefficient (CC), (2) accuracy dependency on T_s_ and P_t_, (3) respiratory pattern dependency, and (4) error for 30% and 70% of the respiration gating for a 5 mm tumor motion for latencies of 300, 500, and 700 ms. Under standard conditions, the Transformer model exhibits the highest accuracy with an RMSE and CC of 0.1554 and 0.9768, respectively. An increase in T_s_ improves accuracy, whereas an increase in P_t_ decreases accuracy. An evaluation of the regularity of the respiratory signals reveals that the lowest predictive accuracy is achieved with irregular amplitude patterns. For 30% and 70% of the phases, the average error of the three models is <1.4 mm for a latency of 500 ms and >2.0 mm for a latency of 700 ms. The prediction accuracy of the Transformer is superior to LSTM and Bi-LSTM. Thus, the three models have clinically applicable accuracies for a latency <500 ms for 10 mm of regular tumor motion. The clinical acceptability of the deep learning models depends on the inherent latency and the strategy for reducing the irregularity of respiration.

## 1. Introduction

Radiation therapy can achieve high dose conformity with intensity-modulated radiation therapy and particle therapy, which can deliver a prescription dose to the target volume while minimizing undesirable doses near critical organs [1, 2]. However, respiratory movements of the patient can result in the administration of undesired doses to the target volume and nearby organs at risk (OARs) [3–5]. Several studies have shown that patient respiration causes organ movements up to 40.0, 39.0, 23.0, and 10.0 mm for the liver, pancreas, kidney, and prostate, respectively [6, 7].

To reduce the dose delivery uncertainty associated with patient respiration during radiation treatment, various methods and technologies, such as deep inspiration breath-hold (DIBH), chest compression, real-time tracking, and respiratory gating, have been introduced.

The DIBH and chest compression methods can reduce dose uncertainty by physically minimizing the movement of the patient’s chest caused by respiration during both computed tomography (CT) simulation and treatments [8–11]. However, for patients who find it difficult to maintain a breath-hold or sustain a chest compression, DIBH or chest compression is not clinically feasible [12, 13]. The real-time tracking method controls beam irradiation by following the tumor position, detected from X-ray fluoroscopy images taken simultaneously during the treatment. Respiratory phase or amplitude gating is a widely used four-dimensional radiation therapy (4DRT) strategy. It synchronizes treatment beam irradiation with patient respiration, represented by an external surrogate, and delivers a beam only at the planned respiration phases (amplitudes). A respiratory-gated treatment plan is developed, as follows. First, the beam delivery phases (amplitudes), such as 30–70% phases (amplitudes) of CT images, are selected from 10 time-resolved phases (amplitudes) of four-dimensional (4D) CT images. Second, the target and OARs are delineated in each of the selected phases (amplitudes) from the CT images, and a dose distribution is computed by average intensity projection (AIP) or maximum intensity projection. Finally, the radiation beam is delivered when the patient’s respiration reaches and is within the selected phases (amplitudes). Although the respiratory gating technique may extend treatment time, it promises an improvement in treatment outcomes and reduces the probability of complications [14, 15].

From a technical point of view, an important prerequisite of precise respiratory-gated radiation therapy in the aforementioned methods is compensating for any system latency of the beam control or modulation system. The system latency is the time delay between the instructed and the actual beam on/off during respiratory-gated radiation therapy. Medical linear accelerators (linacs) have system latencies ranging from 300 ms to 800 ms; the Elekta (Stockholm, Sweden) linacs have latencies of 300–800 ms, and the Varian (Crawley, United Kingdom) linacs have latencies of 300–500 ms [16–18]. The system latency can cause position errors to the target and OARs of up to 7.6 mm [19]. Various mathematical models, such as the autoregressive moving average model (ARIMA) [20, 21], sinusoidal model [22, 23], and Kalman filter [24, 25], have been used to predict respiratory signals. Moreover, recent studies based on machine and deep-learning models demonstrated that the prediction accuracy of such models was superior to that of mathematical models in predicting time series data, and that they resulted in a 150% accuracy improvement [26].

Among various deep-learning models, the recurrent neural network (RNN) is designed to be suitable for time sequence data [27] and has been used with mathematical filters for respiration data prediction [28]. However, the RNN exhibited a deficiency in vanishing or exploding gradients problem [29, 30]. To resolve this deficiency, long short-term memory (LSTM) was introduced with the gradient clipping method by a forget-gate [31]. LSTM is characterized by a three-gate architecture that stores long-term memory and performs well on long time-series data [32]. Lin *et al*. [33] applied LSTM to respiration data acquired by a real-time patient monitoring (RPM) system and demonstrated good performance by optimizing the hyperparameters of the model. A bidirectional long short-term memory network (Bi-LSTM), another RNN variant, was developed to enhance prediction accuracy by using both directions of information on time sequence data [34]. Wang *et al*. [35] reported the superior performance of a seven-layer Bi-LSTM over a mathematical model (autoregressive integrated moving average) and an artificial neural network model (adaptive boosting and multi-layer perceptron neural network) in predicting respiration with a 400 ms latency of the CyberKnife robotic radiosurgery system (Accuray, Sunnyvale, CA. USA). However, comparing the performance of Bi-LSTM with LSTM is somewhat controversial because a model based on Bi-LSTM achieves a superior performance with stock market prediction data [36], whereas a model based on LSTM has better accuracy in research on reservoir inflow forecasting [37]. Another deep-learning algorithm that is suitable for time sequence data is the Transformer. The Transformer is based on an attention mechanism approach, but without recurrent layers [38]. It has the potential to achieve a higher prediction accuracy for time-varying patient respiratory signals [39, 40].

These state-of-the-art deep learning algorithms can be used to realize accurate 4DRT technology. However, none of the studies thus far have evaluated these three models using the same set of clinical data. Moreover, only statistical measures, such as the mean absolute error and root mean square error (RMSE), have been provided as performance metrics in previous studies, thereby limiting clear understanding of the relevant error associated with each model in clinical practice.

Therefore, the performances of LSTM, Bi-LSTM, and the Transformer were evaluated for clinical respiratory signals acquired from patients undergoing proton therapy. In addition, the associated tumor targeting error in gated radiation therapy was measured for various machine latency models, and its clinical applicability was evaluated.

## 2. Materials and methods

### 2.A. Patient respiratory signal data

The data used in this study consisted of 540 respiration signals obtained from 442 patients, who received proton therapy for liver, lung, and breast cancer treatments. The patients took CT simulation with guided free-breathing and were trained in advance by a medical physicist to ensure regular respiratory signals could be obtained using an in-house developed respiration guiding system. Respiration signals were recorded during CT simulation using a respiration gating system (AZ-733VI, Anzai Medical Co. Ltd, Tokyo, Japan). The respiration gating system continuously recorded the respiratory pattern by measuring the distance from the self-emitting laser source to the body surface of the patient at 20 Hz [41]. The recorded time series of the patient’s respiratory signal ranged from 84.35 to 272.50 s, with an average recording time of 145.26 s. This was a retrospective study of patients who received proton therapy. The patient’s respiratory data were recorded from January 01, 2020 to December 31, 2021. The study protocol was approved by the Institutional Review Board of Samsung Medical Center (IRB number 2019-10-159). All respiratory data were fully anonymized before they were accessed.

In addition, the actual target motion in the 4DCT of the patients was analyzed for clinical evaluation. In the anterior-posterior direction, the mean tumor motion was 6.53 mm and the standard deviation was 3.70 mm. In the superior-inferior direction, the mean tumor motion was 11.42 mm and the standard deviation was 8.28 mm. According to the recommendation of the American Association of Physicists in Medicine (AAPM) task group 76a (TG-76a), the maximum tumor motion caused by respiratory motion was limited to 5.0 mm (peak to peak) for external-beam radiation therapy [42].

### 2.B. Data preparation

**Fig 1:**
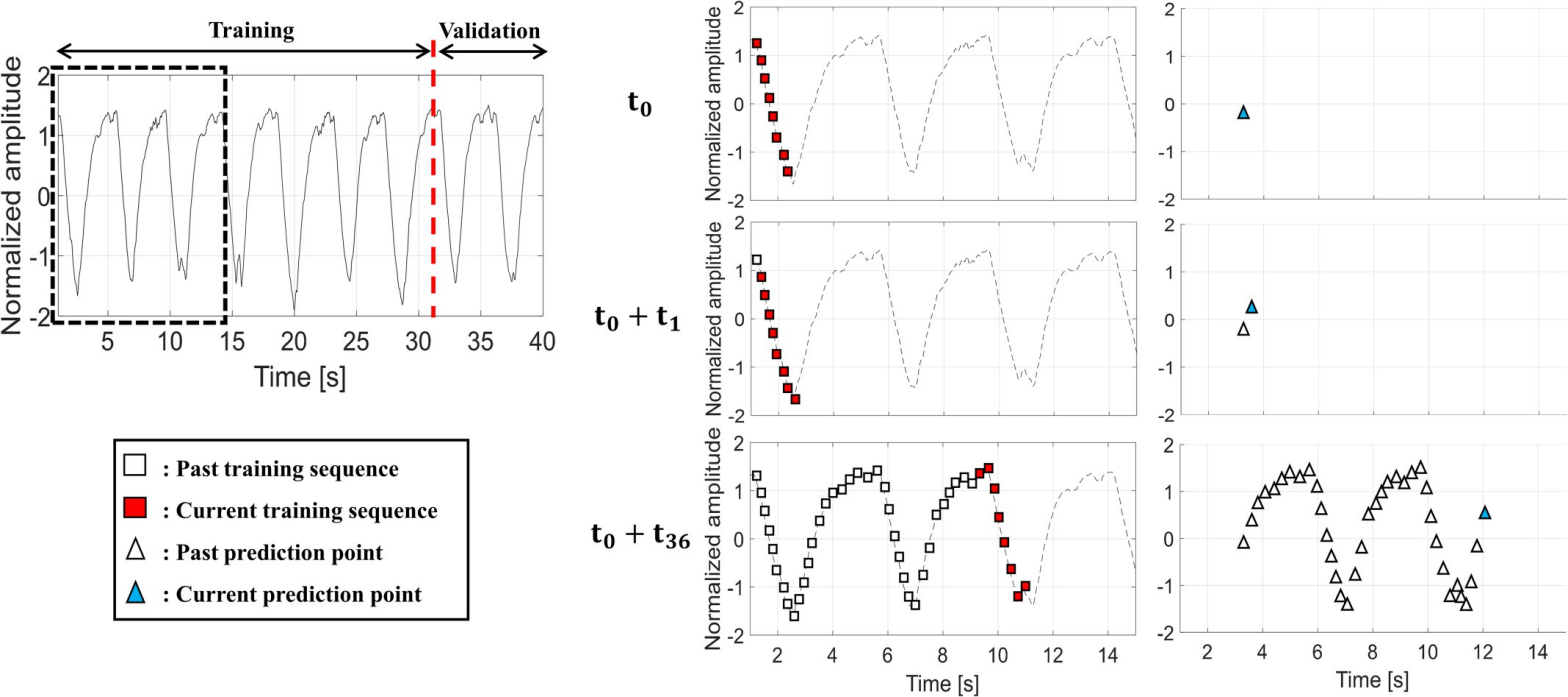
Process of learning a respiratory signal prediction model using deep learning.

Of the total 540 respiration data, 480 respiration data were used as training and validation data, and the remaining 60 respiration data were assigned to the test dataset. Each signal of the 480 respiration data was divided into training and validation data for the deep learning models in a 7:3 ratio (Fig 1).

The input of a deep-learning model predicting the respiratory signal was a training sequence (T_s_), and the output of the model was the prediction point (P_t_) far away from the last point in the T_s_ by the system latency. An example of input and output for the prediction model is shown in Fig 1. In Fig 1, if t = t_0_ and the T_s_ is eight time-points, the sequence consisting of eight points was used as an input and the point P_t_ was predicted by the network. When t = t_0_ + t_n_, the start point of T_s_ moved forward by t_n_; furthermore, P_t_ moved forward by t_n_. In the numerical experiments, the standard condition was set to T_s_ = 1000 ms and P_t_ = 500 ms.

In the case of preprocessing procedures, Z-score normalization was performed to match the baseline of the patient’s respiration signal and to quickly converge the deep learning model [43]. The Savitzky–Golay finite impulse response smoothing (S–G) filter can improve precision without distorting the signal trend [44]. The S–G filter was applied only to the output of the training and test data in the postprocessing step while raw respiratory signals were entered to network. AZ-733VI, a respiration measurement device, had no real scale value because respiration was measured using the phase gating method (there is no unit). Therefore, the quantity can be interpreted as a normalized amplitude.

To analyze the effect of the regularity of the respiratory pattern on the prediction accuracy, the respiration data were classified into four different groups: patterns with regular periods, patterns with irregular periods (type 1), patterns with irregular amplitude (type 2), and patterns with irregular periods and amplitudes (type 3). We defined a respiratory irregularity value using the mean of the standard deviation (STD) in the peaks and the valleys (Eq 1) [45].


$$Irregularity\;value=\frac{STD(peaks)+STD(valleys)}{2}.$$ (1)

The amplitude irregularity was computed using the mean value of the STDs of the amplitudes at the peaks and valleys. For phase irregularity, the periods were computed from the peak and valley times in the signal. The phase irregularity values were computed using the mean of the STDs of the peak-to-peak periods and valley-to-valley periods. After computing both types of irregularity values for all signals, the 48 signals with high amplitude irregularity values were assigned to the type 1 group, and 48 signals with high phase irregularity values were include in the type 2 group. After summation of both types of irregularity values, the 48 signals with high summation values were assigned into the type 3, and 48 signals with low values were assigned to the regular type.

### 2.C. Deep learning models for respiratory signal prediction

**Fig 2:**
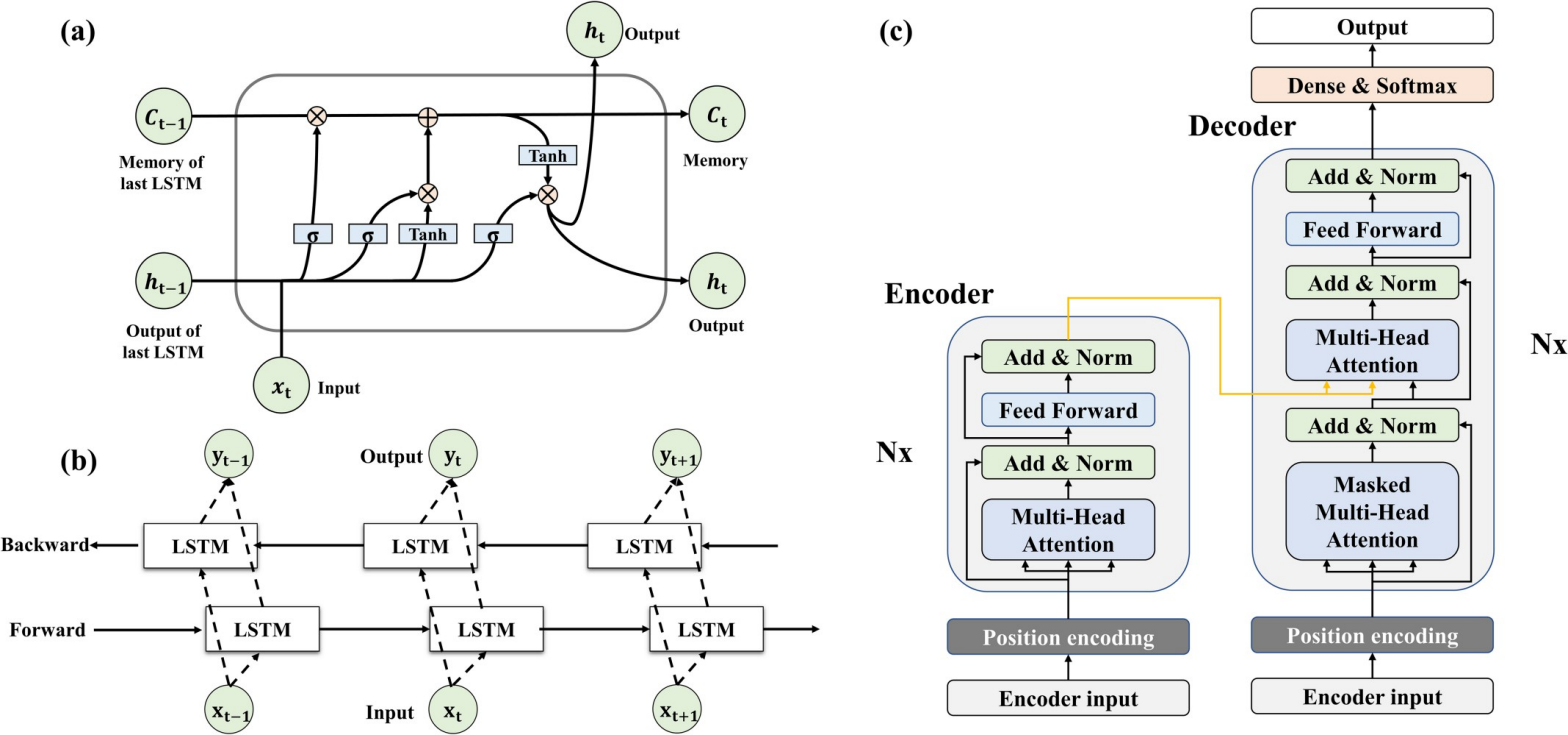
Network structures of the deep learning models used for respiration prediction. (a) Long-short term memory (LSTM): calculation using a forget gate based on RNN, (b) Bidirectional-LSTM: calculations using LSTMs in the backward and forward directions, (c) Transformer: iteratively computes N encoders and decoders.

This section described the three deep learning model characteristics, and the structure of each model is presented in Fig 2.

### 2.C.1. LSTM

The LSTM structure is based on an RNN. LSTM is composed of a forget gate, input gate, and output gate. The forget gate (*f*_*t*_) uses the previous LSTM output (*h*_*t*−1_) and the current input (*x*_*t*_) to determine how much of the previous cell state (*C*_*t*−1_) information is to be maintained (Eq 2). The value of *f*_*t*_ is between 0 and 1. If the value of the *f*_t_ gate is 0, *C*_*t*−1_ is not used; if it is 1, all *C*_*t*−1_ information is used in Eq 5.

The input gate decides whether to add new information to the current cell state (*C*_t_) by using *h*_*t*−1_ and *x*_*t*_ (Eq 4). The input gate consists of two layers: a layer (*i*_*t*_) that selects the values to be updated using a *σ* (Eq 3) and a layer (${\tilde{C}}_{t}$) that creates a new candidate value vector using the hyperbolic tangent function (*tanh*) (Eq 4).

To update the current cell state (*C*_*t*_), each element-wise product of the vectors at *f*_*t*_ and *C*_*t*−1_ and *i*_*t*_ and ${\tilde{C}}_{t}$ are calculated, and the two resulting values are added (Eq 5).

The output gate (*O*_*t*_) determines the part of *C*_*t*_ to be updated using *h*_*t-1*_ and *x*_*t*_ through σ (Eq 6). Output (*h*_*t*_) is calculated by taking *C*_*t*_ to *tanh*, mapping a value between –1 and 1, and determining *O*_*t*_ and the element-wise product of the vectors (Eq 7).


$$f_{t}=\sigma (W_{f}∙[h_{t-1},x_{t}]+b_{f}),$$ (2)

$$i_{t}=\sigma (W_{c}∙[h_{t-1},x_{t}]+b_{c}),$$ (3)

$${\tilde{C}}_{t}=tanh(W_{c}∙[h_{t-1},x_{t}]+b_{c}),$$ (4)

$$C_{t}=f_{t}⊙C_{t-1}+i_{t}⊙{\tilde{C}}_{t},$$ (5)

$$O_{t}=\sigma (W_{o}∙[h_{t-1},x_{t}]+b_{o}),$$ (6)

$$h_{t}=O_{t}⊙tanh(C_{t}),$$ (7)

### 2.C.2. Bi-LSTM

Bi-LSTM has been widely used for natural language translation. Bi-LSTM is a variant of LSTM that uses bidirectional information. It adds information in a direction opposite that of LSTM. The Bi-LSTM output (*y*_*t*_) calculation multiplies and adds the ${\vec{h}}_{t}$ and ${\overset{↼}{h}}_{t}$ outputs of the forward and backward LSTMs, respectively (Eq 8).


$$y_{t}=W_{{\vec{h}}_{y}}{\vec{h}}_{t}+W_{{\overset{↼}{h}}_{y}}{\overset{↼}{h}}_{t}+b_{y}.$$ (8)

### 2.C.3. Transformer

The Transformer is a model that is implemented using the attention mechanism. To achieve computational efficiency, the Transformer uses only multi-head attention mechanisms without convolutional layers and reclusive structures in encoders and decoders. The details of the Transformer are described in the original paper [38].

The Transformer encoders are composed of an input layer, four identical encoder layers, and a position-encoding layer with cosine functions. The identical encoder layer has a multi-head attention layer and a fully connected feed-forward network. The multi-head attention layer is a core structure of the Transformer that can be described as mapping a query (Q), key (K), and value (V) (Eq 9). Technically, these three entities were optimized during the training procedures.

$$Attention(Q,K,V)=softmax(\frac{QK^{T}}{\sqrt{d_{k}}})V,$$ (9)

where K^T^ is the transpose of K, and $\sqrt{d_{k}}$ is the dimension of Q and K. The role of the multi-head attention layer was to allow the model to jointly obtain information from different representation subspaces at different positions. The output of the multi-head attention was passed into the fully connected feed-forward network, which consists of two linear transformations with a rectified linear unit activation function. Subsequently, the output of the fully connected feed-forward network was followed by layer normalization and a residual connection with a decoder.

The decoder consists of an input layer, four identical decoder layers, and an output layer. In the case of the decoder, a third sublayer was inserted into the two sub-layers in each identical decoder layer. The third sublayer was a masked multi-head attention layer that could prevent self-attention. Based on the original paper, we used look-ahead masking and one-position offset between the decoder input and target output in the decoder to ensure that the prediction of a time-series data point depended only on previous data points. Finally, the output layer mapped the output of the last decoder layer to the target time sequence.

### 2.D. Training of LSTM, Bi-LSTM, and the Transformer for respiratory signal prediction

To train the LSTM, Bi-LSTM, and Transformer to predict a respiratory signal for respiratory-gated radiation therapy, the hyperparameters of each model were determined as follows.

To compare the accuracies of all three models when they achieved their best performances, the LSTM and Bi-LSTM models consisted of 15 hidden layers with a size of 3 nodes, respectively [33]. The Transformer model consisted of eight multi-head layers for the attention mechanism, six encoder layers, and six decoder layers [38]. The number of the hidden layers and multi-head layers were empirically determined.

Adaptive moment estimation [45] was used for the three deep-learning models, with a learning rate of 0.0001 and weight decay of 0.0002. The two beta parameters, which were default parameters relevant to the running average of the gradient in the adaptive moment optimizer, were set to *β*_1_ = 0.9 and *β*_2_ = 0.999. The batch size for the training was set to 300. Model training was performed for 100 epochs, and the best validation performance model was used for evaluation. The training procedure was performed using an NVIDIA GeForce 2080Ti graphic processing unit on Pytorch 1.5.1. The best model was updated when the current validation loss was smaller than the previous validation loss obtained during the training procedure.

### 2.F. Evaluation

To evaluate the performance of the models, the difference between the actual and predicted respiratory signals was quantitatively assessed by computing the RMSE (Eq 10), and Pearson’s correlation coefficient (CC, Eq 11).

$$RMSE=\sqrt{\sum_{i=1}^{n}\frac{{\left({\hat{y}}_{i}-y_{i}\right)}^{2}}{n}},$$ (10)

where *y*_*i*_ is the actual respiratory signal and ${\hat{y}}_{i}$ is the predicted respiratory signal.

The CC was used to analyze the linear relationship between the two continuous variables. The CC takes values in the interval [–1.0, 1.0]. If the value of CC is closer to 1.0, on an absolute scale, the correlation between the ground truth and the predicted value is stronger.

$$CC=\frac{\sum \left(y_{i}-\bar{y})({\hat{y}}_{i}-\bar{\hat{y}}\right)}{\sqrt{\sum {\left(y_{i}-\bar{y}\right)}^{2}\sum {\left({\hat{y}}_{i}-\bar{\hat{y}}\right)}^{2}}},$$ (11)

where *y*_*i*_ is the actual respiratory signal; ${\hat{y}}_{i}$ is the predicted respiratory signal; and $\bar{y}$ and $\bar{\hat{y}}$ are the average values of *y*_*i*_ and ${\hat{y}}_{i}$, respectively. In addition, the statistical significance of the differences in the predictions achieved with the three models was analyzed. The P-values of the RMSE and CC of each prediction model were computed using a one-way analysis of variance (one-way AVOVA).

For the comparative study, the prediction accuracy was calculated on the i) standard condition of the T_s_ and P_t_. In addition, we analyzed the ii) effect of the length of T_s_ and P_t_ on prediction accuracy and iii) the effect of the regularity of the respiratory pattern prediction accuracy. Finally, we analyzed the suitability of the three deep learning-based respiratory signal prediction models for respiration-gated radiation therapy to translate the statistical error into clinical measures. For this purpose, the respiration signal was assumed as identical to the tumor motion with an amplitude of 10 mm, and the model prediction error of 30% and 70% of the respiration phases was computed in mm, thereby allowing for evaluation of the clinical applicability of the models.

## 3. Results

### 3.A. Evaluation of the prediction accuracy

**Fig 3:**
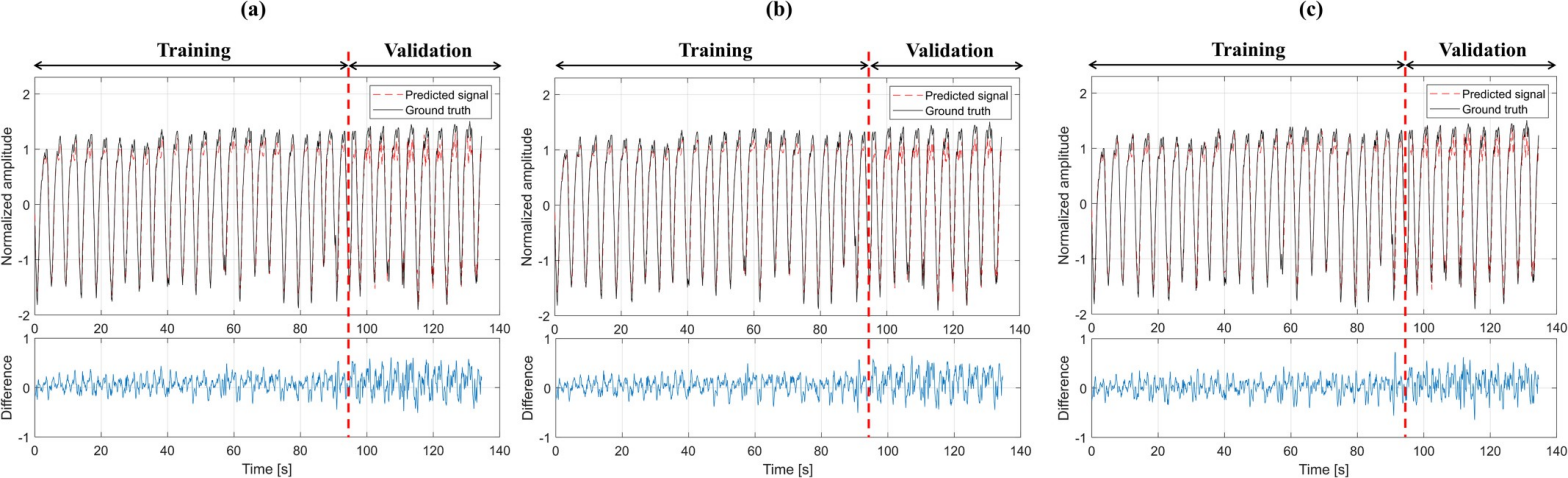
Plots of actual (solid line) and predicted respiratory signals (dotted line). (a) LSTM, (b) Bi-LSTM, and (c) Transformer. The difference is an absolute value (true signal amplitude–predicted signal amplitude).

The prediction accuracy of the respiratory signal was calculated for LSTM, Bi-LSTM, and the Transformer. The results were obtained under the standard conditions of T_s_ = 1000 ms and P_t_ = 500 ms. Examples of the prediction results of LSTM, Bi-LSTM, and the Transformer are shown in Fig 3. The averaged RMSE and CC of the validation and test sets for the three different deep learning models are summarized in Table 1. In the test set for the LSTM, Bi-LSTM, and the Transformer, the RMSEs were 0.1907, 0.1930, and 0.1554, and the CCs were 0.9689, 0.9661, and 0.9768, respectively. According to the data summarized in Table 1, the Transformer exhibited a relatively high performance in the two types of test sets compared with the other respiratory prediction methods. Based on a one-way ANOVA, the P-values were 0.0001 and 0.0002 for the RMSE and CC for the validation set, and 0.0051 and 0.0324 for the RMSE and CC for the test set. All of the P-values were less than 0.05, thereby indicating a significant difference.

**Table 1:** Respiratory signal prediction accuracies of LSTM, Bi-LSTM, and the Transformer.

		RMSE	CC
Validation set	LSTM	0.2501	0.9667
	Bi-LSTM	0.2554	0.9640
	Transformer	**0.2309**	**0.9715**
Test set	LSTM	0.1907	0.9689
	Bi-LSTM	0.1930	0.9661
	Transformer	**0.1554**	**0.9768**

*The values in bold are the high scores in this comparison study

### 3.B. Effect of the training sequence (T_s_) and prediction point (P_t_) on prediction accuracy

**Fig 4:**
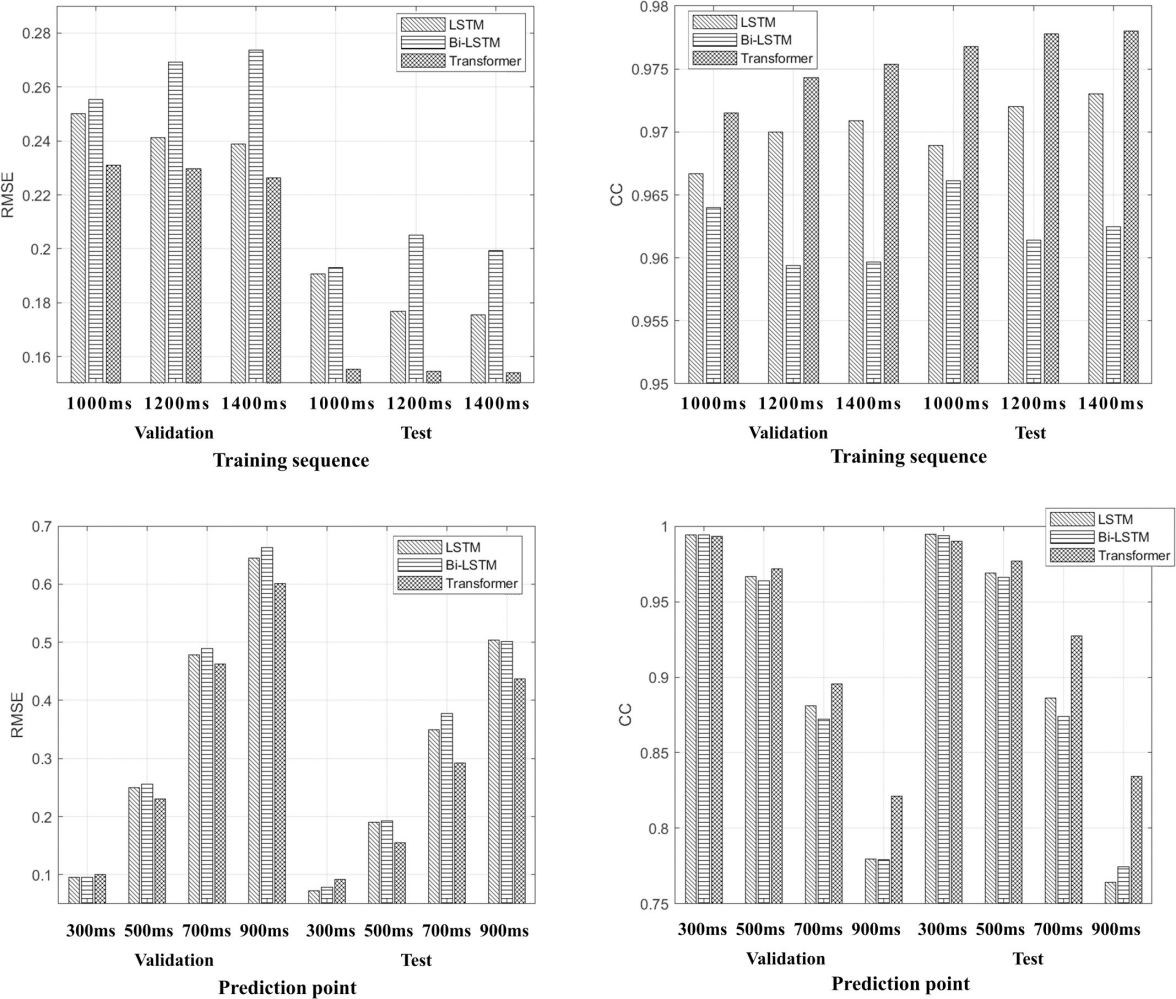
Comparison of the prediction accuracies of LSTM, Bi-LSTM, and the Transformer for different training sequences (T_s_) and prediction points (P_t_).

To analyze the effect of the training data length of T_s_ on prediction accuracy, the prediction accuracy of respiratory signals among the three different models was assessed using three different T_s_: 1000, 1200, and 1400 ms. The P_t_ was fixed at 500 ms. The RMSE and CC were calculated for three different T_s_ values. The results are summarized in Fig 4.

In the validation set with 1200 ms of T_s_, the RMSEs were 0.2412, 0.2692, and 0.2398 for LSTM, Bi-LSTM, and Transformer, respectively. In the test set with 1200 ms of T_s_, the RMSEs were 0.1769, 0.2053, and 0.1559 for LSTM, Bi-LSTM, and the Transformer, respectively. As shown in Fig 4, the Transformer exhibited a relatively high prediction accuracy for the three T_s_ values.

To analyze the effect of the length of P_t_ on prediction accuracy, T_s_ was fixed at 1000 ms, and P_t_ was set as 300, 500, 700, and 900 ms. The RMSE and CC values were calculated and are summarized in Fig 4. As the length of P_t_ increased, the performance of all models reduced. At P_t_ = 300 ms, the prediction errors of the three models were equivalent. However, for P_t_ longer than 500 ms, the performance of the Transformer was higher than that of the other models.

### 3.C. Evaluation of the prediction accuracy for different respiration patterns

**Fig 5:**
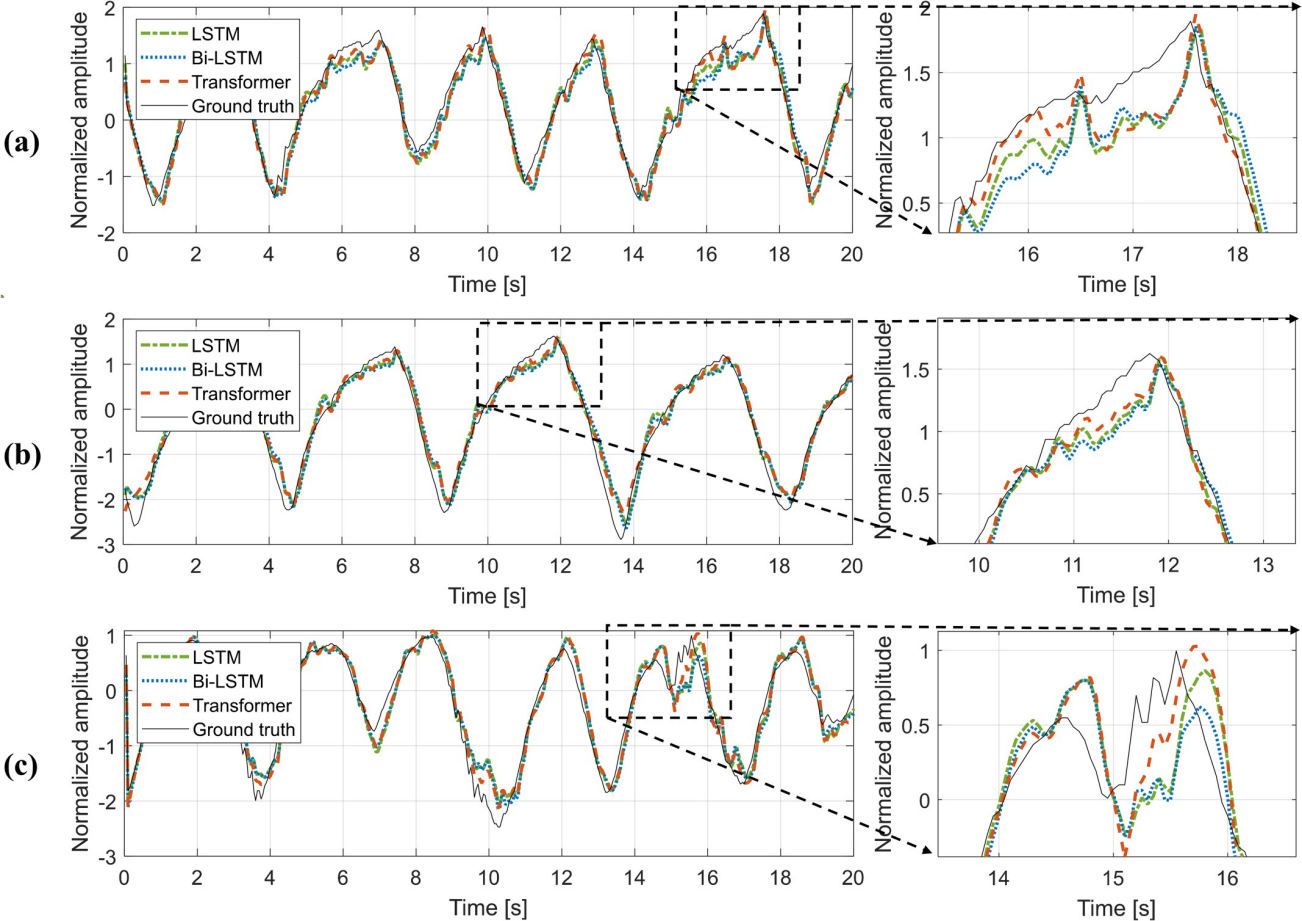
Actual and predicted respiratory signals for three different patterns of irregular respiration. (a) irregular pattern with periods, (b) irregular pattern with amplitude, and (c) irregular pattern with periods and amplitude.

The RMSE and CC were calculated and summarized for each irregular respiratory pattern (Table 2). Herein, the accuracy for regular respiration is presented as a reference. For the irregular pattern of type 3, the RMSEs were 0.2882, 0.2837, and 0.2773 for LSTM, Bi-LSTM, and the Transformer, respectively. In these quantitative assessments, the Transformer exhibited the highest prediction accuracy for the three groups with irregular respiratory patterns. When compared to the accuracy for regular signals, the highest increment of RMSE and the highest decrement of CC were observed for signals with an irregular amplitude pattern. The prediction deviated in regions where respiration signals were not smooth, and in particular, respiration amplitude irregularity was observed (Fig 5).

**Table 2:** Accuracy evaluation of the LSTM, Bi-LSTM, and Transformer methods using the RMSE and CC.

	irregular						regular	
	Type 1 (Period)		Type 2 (Amplitude)		Type 3 (Period and amplitude)			
	RMSE	CC	RMSE	CC	RMSE	CC	RMSE	CC
LSTM	0.2601	0.9636	0.2733	0.9547	0.2882	0.9555	0.2501	0.9667
Bi-LSTM	0.2602	0.9624	0.2802	0.9517	0.2937	0.9555	0.2554	0.964
Transformer	**0.2436**	**0.9696**	**0.261**	**0.9613**	**0.2773**	**0.9613**	**0.2367**	**0.9717**

### 3.D. Analysis of clinical feasibility

To analyze the clinical feasibility of the deep-learning methods for respiration-gated radiation therapy, we assumed that the maximum tumor motion caused by respiration motion was limited to 10.0 mm.

The treatment plan configured with respiratory-gated radiotherapy is developed typically based on the AIP. CT images were calculated from 4D CT images by averaging or accumulating the HU number at each voxel from the CT images of selected respiration phases, such as 40–60% or 30–70% of the phases among 10 phases of respiration. Thus, each full respiration cycle of the obtained signals was divided into 10 phases, and 30% and 70% of the phases were selected as beam on/off phases, respectively. Because the predicted and actual respiration signals can have different peak positions, the time points of the 30% and 70% of the phases of the two respiration signals were different. Thus, the amplitude difference between two signals at the phases in the predicted signals was computed as an error. For the numerical validation experiment, T_s_ was set to 1000 ms. In the case of P_t_, three different latencies (300, 500, and 700 ms) were used.

**Fig 6:**
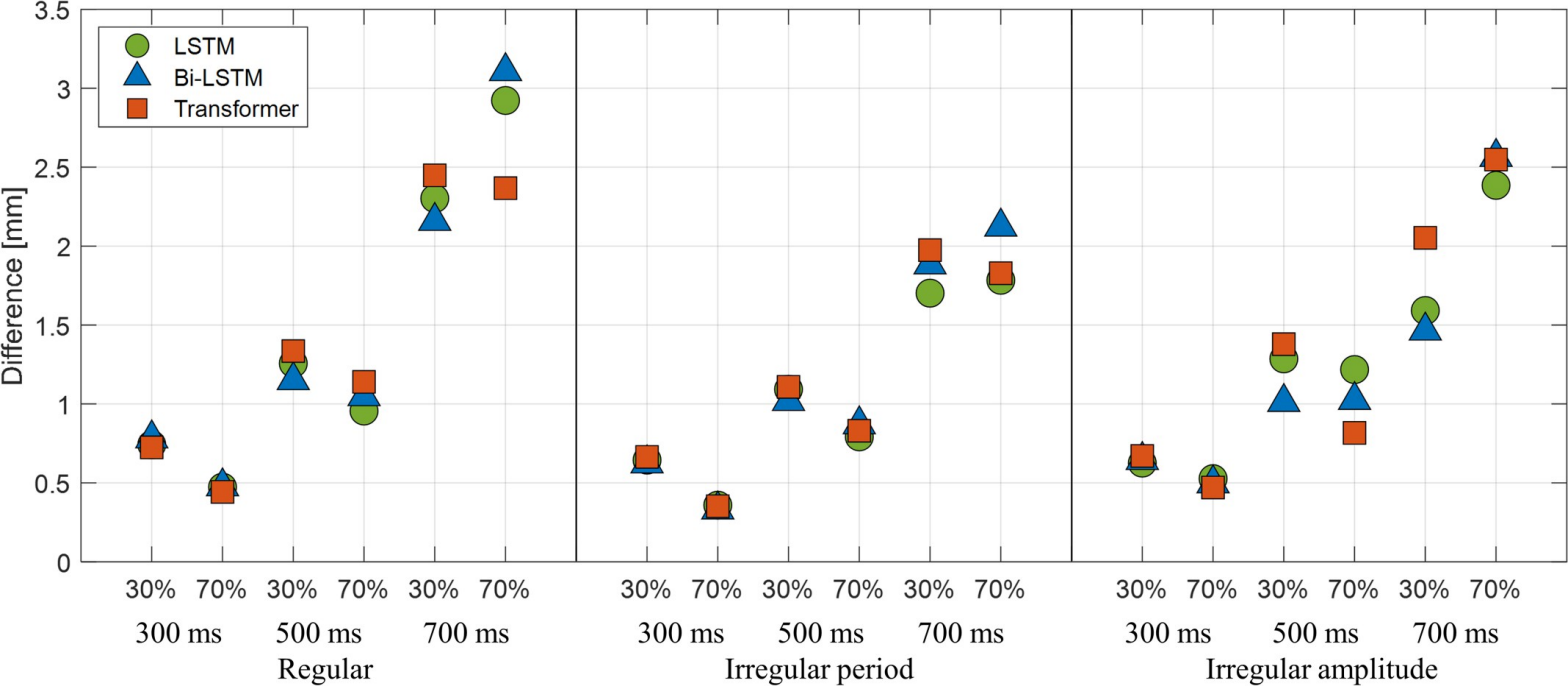
Average error results in millimeters. At 30% and 70% phases for regular and irregular patterns with three different prediction points: 300, 500, and 700 ms.

**Fig 7:**
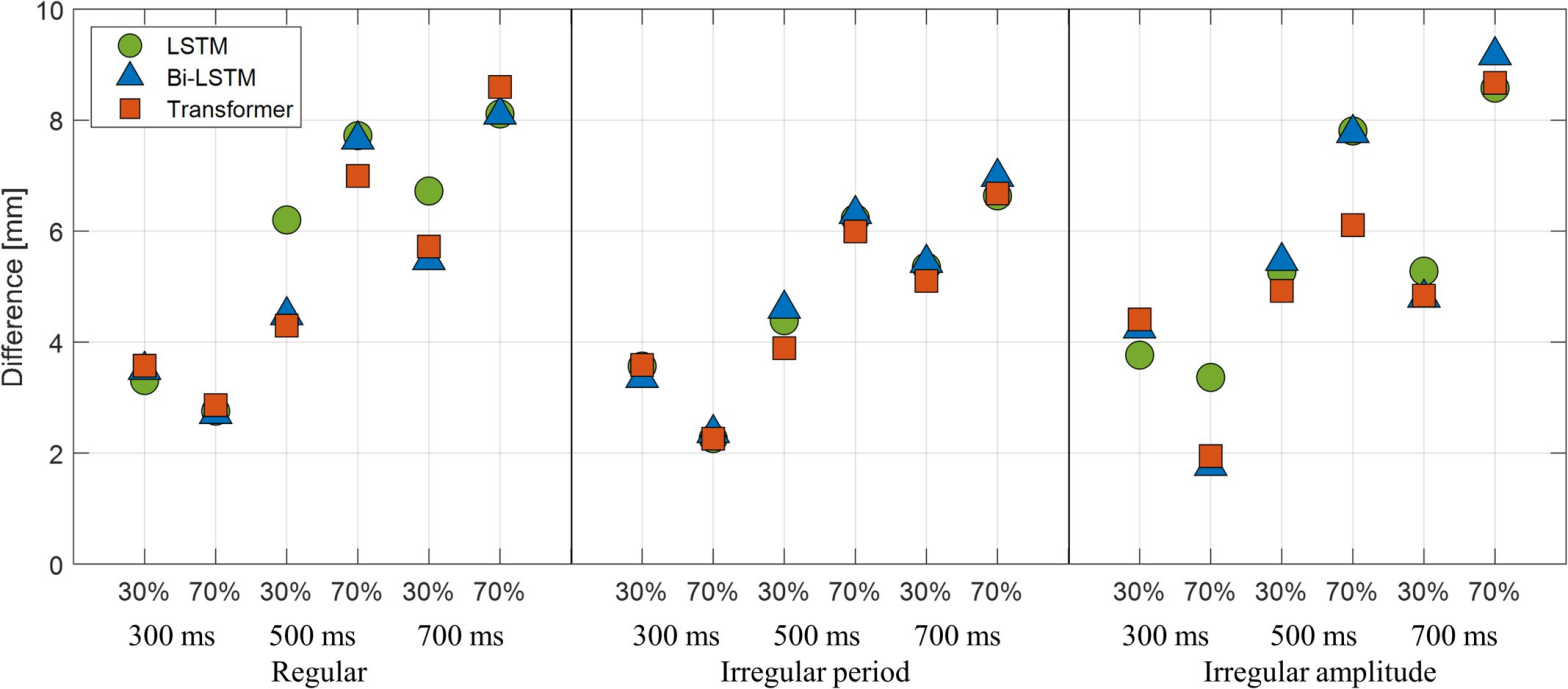
Maximum error results in millimeters. At 30% and 70% phases for regular and irregular patterns with three different prediction points: 300, 500, and 700 ms.

Ten representative cases for regular and irregular period (type 1) and irregular amplitude patterns (type 2) of motion are presented in Figs 6 and 7, respectively. The average errors of the three models were less than 0.78 mm and 1.38 mm with a latency of 300 ms and 500 ms, respectively. However, the average error was higher than 2.0 mm for 700 ms of latency. The maximum error of the prediction was 4.41 mm, 7.80 mm, and 9.17 mm at P_t_ values of 300, 500, and 700 ms, respectively.

## 4. Discussion

For precise 4DRT with a respiratory-gated system, a model that can compensate for the latency of the beam delivery system is essential. Thus, we compared the respiratory signal prediction performance of three deep learning-based prediction models: LSTM, Bi-LSTM, and Transformer. The Transformer achieved the best prediction accuracy under standard conditions in both the validation and test sets. In the test set, the performances of the LSTM, Bi-LSTM, and Transformer had CC values of 0.9689, 0.9661, and 0.9768, respectively.

Additionally, we analyzed the effects of T_s_ and P_t_ on the prediction accuracy because the beam on/off latency differs depending on manufacturer, motion detection device, and linac model. As the training length of T_s_ increased, the amount of information supplied to the model increased; thus, the prediction accuracy improved for both LSTM and the Transformer, but not for Bi-LSTM. In the case of P_t_, as P_t_ increased, the prediction accuracy reduced for all three models. Therefore, the prediction accuracy was affected by the training data length (T_s_) and the time distance to the prediction (P_t_.)

With regard to the prediction accuracy, when the beam on/off latency of a linac was less than or equal to 300 ms, LSTM, Bi-LSTM, and the Transformer exhibited similar performances, with CC values of 0.9943, 0.9942, and 0.9934, respectively. However, when the beam on/off latency exceeded 500 ms, the prediction achieved with the Transformer was superior to that of LSTM and Bi-LSTM. In particular, when the beam on/off latency was 900 ms, LSTM, Bi-LSTM, and the Transformer exhibited significant performance differences, with CC values of 0.7797, 0.7794, and 0.8212, respectively. In the analysis of T_s_ and P_t_, Transformer outperformed LSTM and Bi-LSTM.

Based on clinical observations, numerous patients do not have perfectly regular respiration patterns. Respiration patterns are affected by daily conditions, stress level, and the severity of the disease. Thus, the prediction accuracy of the models is important in the case of irregular respiration patterns. A comparison of the irregular respiration signals revealed that irregularities in respiration amplitude have the greatest impact on prediction accuracy. Therefore, when training and preparing a patient for respiratory-gated radiation therapy, efforts should be made to minimize variation in the patients’ respiration amplitude.

Among the RNN-based deep learning models, Bi-LSTM perform better than LSTM in stock market prediction studies [36]; however, LSTMs are more suitable for reservoir inflow prediction studies [37]. In the analysis performed in this study, the predictive accuracy of LSTM was better than that of Bi-LSTM, although the difference in the evaluation metrics was small. There is a difference in performance between LSTM and Bi-LSTM depending on the data used; therefore, verification is required when predicting respiration using LSTM and Bi-LSTM.

Because the accuracy evaluation in this study deals with the average errors in predicting respiratory signals, it does not clearly present the associated errors in clinical practice. To evaluate the clinical impact of a predictive model for gated radiation therapy, three representative patterns of respiration signals were used to mimic tumor movement, the patient’s respiration was managed to limit tumor motion to 10 mm, and the prediction error was calculated in millimeters. The results in Fig 6 reveal that when the delay time was 300 ms, all three models exhibited an average error of less than 0.78 mm, which is considered acceptable for patient treatment. The Transformer exhibited the lowest error in three out of six in the average error evaluations, but the maximum observed error was 4.41 mm during motion with an irregular amplitude. When the delay time was 500 ms, the average error was less than 1.38 mm, the maximum value of the error increased in all models, and the maximum error of 7.80 mm was exhibited by LSTM. The Transformer exhibited the lowest maximum error among the three respiratory signal patterns. When the delay time was 700 ms, the maximum error was 9.17 mm, and the average error was approximately 3.11 mm. Therefore, with the beam delivery system with delay times of 300 and 500 ms, the respiratory prediction model can achieve an acceptable performance (< 1.38 mm on average for 10 mm of tumor motion), and with the beam delivery system having a beam latency of 700 ms, the respiratory prediction model potentially generates an error larger than 3.11 mm on average for 10 mm of tumor motion.

Although the movement of the external respiration signal was assumed to be quantitatively identical to the movement of the tumor, such a scenario rarely occurs in real clinical situations. Nevertheless, the hypothesis enables us to estimate and determine the error range when applying deep learning-based prediction models to compensate for the beam delay time of a radiation therapy device. As the performances of the models were compared based on the relative error using a normalized respiration signal, quantitative evaluation in clinical practice was necessary. In a future study, we plan to use one-dimensional respiratory signals to predict three-dimensional tumor motion over time through an analysis of respiratory signals and tumor motion.

## 5. Conclusion

We successfully evaluated the clinical feasibility of LSTM, Bi-LSTM, and Transformer models for respiratory signal prediction. Among the deep learning-based models, the Transformer model was superior to the LSTM and Bi-LSTM models. Prediction accuracy was found to be affected by the training data length and the time distance to the prediction, and was considerably affected by irregular amplitude patterns. Thus, the feasibility of using a respiratory prediction deep learning model for a clinical application depends on the beam on/off latencies of the radiation therapy equipment. In addition, patient respiration management strategies are fundamentally important factors in 4DRT.
